# Evidence of Recombination and Genetic Diversity in Human Rhinoviruses in Children with Acute Respiratory Infection

**DOI:** 10.1371/journal.pone.0006355

**Published:** 2009-07-27

**Authors:** Ting Huang, Wei Wang, Mael Bessaud, Peijun Ren, Jun Sheng, Huajie Yan, Jing Zhang, Xin Lin, Yongjin Wang, Francis Delpeyroux, Vincent Deubel

**Affiliations:** 1 Institut Pasteur of Shanghai, Chinese Academy of Sciences, Shanghai Institute of Biological Sciences, Unit of Emerging Viruses, Shanghai, People's Republic of China; 2 Institut Pasteur, Unit of Biology of Enteric Viruses, Department of Virology, Paris, France; 3 Shanghai Nanxiang Hospital, Shanghai, People's Republic of China; Comprehensive AIDS Reseach Center, China

## Abstract

**Background:**

Human rhinoviruses (HRVs) are a highly prevalent cause of acute respiratory infection in children. They are classified into at least three species, HRV-A, HRV-B and HRV-C, which are characterized by sequencing the 5′ untranslated region (UTR) or the VP4/VP2 region of the genome. Given the increased interest for novel HRV strain identification and their worldwide distribution, we have carried out clinical and molecular diagnosis of HRV strains in a 2-year study of children with acute respiratory infection visiting one district hospital in Shanghai.

**Methodology/Findings:**

We cloned and sequenced a 924-nt fragment that covered part of the 5′UTR and the VP4/VP2 capsid genes. Sixty-four HRV-infected outpatients were diagnosed amongst 827 children with acute low respiratory tract infection. Two samples were co-infected with HRV-A and HRV-B or HRV-C. By comparative analysis of the VP4/VP2 sequences of the 66 HRVs, we showed a high diversity of strains in HRV-A and HRV-B species, and a prevalence of 51.5% of strains that belonged to the recently identified HRV-C species. When analyzing a fragment of the 5′ UTR, we characterized at least two subspecies of HRV-C: HRV-Cc, which clustered differently from HRV-A and HRV-B, and HRV-Ca, which resulted from previous recombination in this region with sequences related to HRV-A. The full-length sequence of one strain of each HRV-Ca and HRV-Cc subspecies was obtained for comparative analysis. We confirmed the close relationship of their structural proteins but showed apparent additional recombination events in the 2A gene and 3′UTR of the HRV-Ca strain. Double or triple infections with HRV-C and respiratory syncytial virus and/or bocavirus were diagnosed in 33.3% of the HRV-infected patients, but no correlation with severity of clinical outcome was observed.

**Conclusion:**

Our study showed a high diversity of HRV strains that cause bronchitis and pneumonia in children. A predominance of HRV-C over HRV-A and HRV-B was observed, and two subspecies of HRV-C were identified, the diversity of which seemed to be related to recombination with former HRV-A strains. None of the HRV-C strains appeared to have a higher clinical impact than HRV-A or HRV-B on respiratory compromise.

## Introduction

Human rhinoviruses (HRVs) are a highly p revalent cause of the acute respiratory infection (ARI) defined as the common cold [1], [2], [3], which is frequently associated in children with bronchitis, bronchiolitis, wheezing, pneumonia, asthma and otitis [4], [5], [6], [7], [8], [9]. HRVs are classified in genus Enterovirus (HEVs) in family Picornaviridae [10]. HRVs are non-enveloped, single-stranded, positive-sense RNA viruses of approximately 7200 nt, composed of a 5′ untranslated region (UTR), followed by a long open reading frame coding for capsid proteins VP4, VP2, VP3 and VP1, and seven non-structural proteins 2A, 2B, 2C, 3A, 3B, 3C and 3D, and terminated by a short 3′UTR and poly A tract.

More than 100 serotypes of HRV are known, which have been classified into two species, HRV-A and HRV-B, according to comparative alignment of nucleotide fragments of VP1 [11], [12], VP4/VP2 [13] and 5′UTR [14], [15], and more recently, on complete genome nucleotide sequences [16]. Moreover, some genomic sequences have been found not to cluster with HRV-A and HRV-B species, which suggests the existence of other species (HRV-C and HRV-D) [16]. A new species of HRV-C was recently identified worldwide by comparative analysis of VP4 or VP4/VP2 genes [7], [17], [18], [19], [20], [21] and 5′UTR [14], [15]. However, discrepancies have appeared in the classification of some of the new HRV-A or HRV-C strains, depending on the size and location of the nucleotide sequence in the viral genome and on the phylogenetic methods used for direct analysis of HRV sequences [3], [7], [14], [15], [17], [18], [20], [21], [22], [23].

These recent data underline the lack of knowledge about the biodiversity of HRV strains and their worldwide distribution [14], [17]. Moreover, little is known about the characteristics and diversity of HRVs circulating in a given area in a short period of time. In the present study, we looked for HRVs in a 2-year collection of nasopharyngeal swabs (NPSs) of children with ARI visiting a district hospital in Shanghai, and compared sequences in two regions previously defined for genetic classification of HRV serotypes [13], [15]. Our study showed a high diversity of HRV species and genotypes, and a prevalence of the novel HRV-C species in NPSs of children with bronchitis and pneumonia. This biodiversity appeared to result partly from recombination events in the 5′UTR, between HRV-C strains and those close or similar to HRV-A species, which led to the suggested classification of HRV-C into at least two subspecies.

## Results

### Identification and typing by phylogenetic analysis of 66 HRVs in NPSs from children with ARI

Eight hundred and twenty-seven samples were collected from a group of children consulting the Shanghai Nanxiang Hospital during a 2-year period, and tested for 17 respiratory viruses using a multiplex RT-PCR (mRT-PCR). Sixty-four samples (7.7%) were positive for HRV, according to the length of the amplified fragment in the VP4/VP2 region visualized on agarose gel (data not shown). A larger fragment of 924 nt, including part of the 5′UTR (starting at nt 163) and the VP4/VP2 genes (ending at nt 1086), was amplified and cloned into plasmid vectors for genetic analysis (Table 1). Only one sample, N1, could not be amplified and was amplified in two steps in the 5′UTR (nt 163–552) and in the VP4/VP2 region (nt 528–1086), respectively. Analyses of different clones for each sample allowed the identification of multiple infections: sample N16 was shown to contain one HRV-A and one HRV-B (N16A and N16B, respectively), while N58 contained one HRV-A and one HRV-C (N58A and N58C, respectively) (see below). In order to further characterize the 66 HRVs identified in the 64 samples, the nucleotide sequences located in the 5′UTR (285 nt) and the VP4/VP2 genes (420 nt) were chosen to allow comparative alignment with sequences of reference serotypes and field strains available in GenBank.

**Table 1:** Degenerate and specific primers used for RNA amplification from clinical samples.

5′–3′ Sequence	Virus/Gene	Start^a^	End^a^	Reference
CAAGCACTTCTGTYWCCCC	P1-1 F	163	181	[15]
GGGACCAACTACTTTGGGTGTCCGTGT	VP4/2 F	528	554	[13]
ACGGACACCCAAAGTAG	P3-1 R	536	552	[15]
GCATCIGGYARYTTCCACCACCANCC	VP4/2 R	1061	1086	[13]
YCCWCCACARTCWCCWGGTTC	2A R	3488	3508	[12]
TWGCHTTTGAYTACWCNGGNTATGA	3D outer F	6396	6420	[35]
ATGATHGCHTATGGDGAYGAYGT	3D inner F	6668	6690	[35]
GGGTCYYTAGTCCATCTGATTGAYTC	3D inner R	6896	6921	This study
GTAIKGYTCYTCWCCATTGTGCCA	3D outer R	6965	6988	*Id*
CCGGGGAAACAGAAGTGCTTGAA	N4 5′race SP2 R	161	183	*Id*
CTCTGCTTAGTAATTGCGCGGGTA	N4 5′race SP1 R	228	251	*Id*
CACCTCTGTGGATAAGCCCACTCAT	N4 VP2 outer F	973	997	*Id*
AGCCCACTCATCCAGAAACATCAG	N4 VP2 inner F	987	1010	*Id*
GGTGAGGGTCCATGTGAACCWGGT	N4 2A outer F	3473	3496	*Id*
GGGTCCATGTGAACCWGGTGACTGT	N4 2A inner F	3478	3502	*Id*
CACGTCATCCCCATATGCGATCA	N4 3D inner R	6669	6691	*Id*
CAATCACGTCATCCCCATATGCGA	N4 3D outer R	6672	6695	*Id*
CTAAATACCCCTTCCTCATTCATCCA	N4 3D outer F	6846	6871	*Id*
CAATCAGATGGACTAGGGACCCAA	N4 3D inner F	6900	6923	*Id*
CCTCGGTGGAAGCCTATTCACACATC	N10 5′race SP2 R	183	208	*Id*
GGGTTAAGGTTAGCCACATTCAGGG	N10,N13 5′race SP1 R	447	471	*Id*
TGGTGGTGGAARTTACCCGATGC	N10,N13 VP2 F	1064	1086	*Id*
CTGGGTATGAAGCAATCACGTC	N10 3D inner R	6686	6707	*Id*
CTGCTGGGGTTATGGTGAGAC	N10 3D outer R	6753	6773	*Id*
CCAGACACTAAATACCCCTTCCTC	N10 3D F	6839	6862	*Id*
TTTGCCCTGGCGGAGCATATCAGC	N13 5′race SP2 R	189	212	*Id*
CCCACATGTGACTGCACTCAAGCA	N13 2A outer F	3332	3355	*Id*
GGTATTACCCAATTAATGTTACCAG	N13 2A inner F	3381	3405	*Id*
GCATATGGGGAYGACGTCRTCTT	N13 3D outer F	6674	6696	*Id*
GCTGGAGTGATTGTAAGACCATA	N13 3D R	6749	6771	*Id*
CAGATGGACTAARGACCCAAT	N13 3D inner F	6904	6924	*Id*

a Nucleotide position relative to HRV16.

W = (A/T),H = (A/T/C),D = (G/A/T),Y = (C/T),K = (G/T), R = (A/G),N = (A/G/C/T).

I:Inosine.

*Id*: this study.

**Figure 1:**
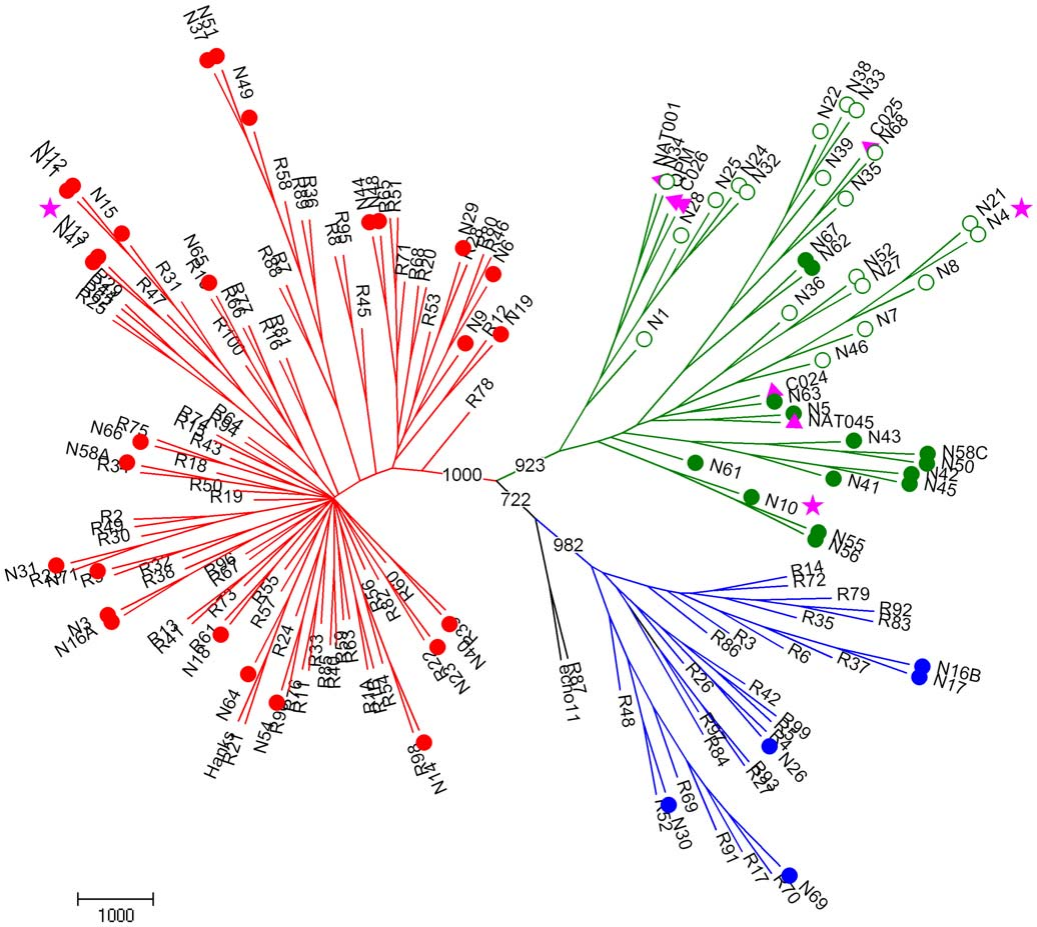
Phylogenetic tree depicting relationships between known and novel HRVs based on VP4/VP2 gene analysis. Grouping of HRV reference serotypes (designated R#), previously published novel HRV strains QPM from Australia (GenBank accession no. E186077), NAT001 and NAT045 from California (GenBank accession no. EF077237 and EF077281), and C024, C025, and C026 from Hong Kong (GenBank accession no. EF582385, EF582386, EF582387) designated by a pink triangle, and viral strains detected in our clinical samples designated N# and by open and filled circles, was based on 420 nt in the VP4/VP2 gene region. Strains fully sequenced in this study are designated by a pink star. HRV-A, HRV-B and HRV-C are drawn with red, blue and green colors, respectively. Strains classified in HRV-Cc and HRV-Ca subspecies are indicated by filled and open circles, respectively. Echo 11 and R87 prototypes are included as outgroups. Tree construction and bootstrap values indicated for each major branch in the tree were determined with PHYLIP package and SEQBOOT, with 1000 replicates.

We first compared and classified Shanghai strains according to their VP4/VP2 sequences. The pairwise nucleotide divergence in the VP4/VP2 region ranged from 0 to 72.2%. Twenty-seven HRVs (40.9%) showed >81% nucleotide identity with the closest HRV-A clusters, and five HRVs (7.6%) showed >88.8% nucleotide identity with HRV-B clusters (Table 2). The remaining 34 strains (51.5%) diverged from HRV-A and HRV-B species by >47.3% in their VP4/VP2 nucleotide sequence (Table 2). These strains showed from 68.3 to 100% nucleotide identity with each other and were related to the recently described HRV-C strains, NAT001 and NAT045, isolated in California, USA [18], C024, C025 and C026 in Hong Kong [20], and QPM in Australia [7], [24] (Fig. 1). Strains N34, N35 and N68 were closely related to the recently identified strains C025 and NTA001 with >95.9% nucleotide identity, whereas the 31 remaining HRV-C strains showed only 74.4–86.4% nucleotide identity with six other recent strains (QPM, NAT001, NAT045, C024, C025 and C026; Table 2), which were classified tentatively as HRV-C species [7], [18], [20], [24]. Classification of the strains into three different species was also demonstrated by construction of a phylogenetic tree using aligned VP4/VP2 sequences (Fig. 1).

**Table 2:** Characterization of 66 clinical HRV strains in clinical specimens based on partial VP4/VP2 gene sequences.

Clinical HRV strain	Nearest	Divergence with the	Species (subspecies^a^)
(% nt identity^b^)	HRV strain	nearest HRV (% nt)	classification
N64	Hanks	5.3	A
N14	R98	5.8	A
N31	R23	6.3	A
N3,N16A (98.8)	R38	6.5	A
N19	R12	7.7	A
N66	R75	8.5	A
N65	R10	9	A
N23	R22	9	A
N13,N47 (98.6)	R47	9.1–9.7	A
N37,N49,N51^c^ (98.1–99)	R58	9.2–10.4	A
N40	R39	9.4	A
N29	R28	9.6	A
N58A	R34	10	A
N71	R9	10.2	A
N54	R90	10.2	A
N11,N12,N15^c^ (99.5–100)	R31	10.5	A
N18	R61	11.9	A
N6	R46	15.3	A
N9	R28	18.9	A
N44,N48^c^ (99.8)	R65	22.1–22.4	A
N26	R4	10.1	B
N69	R70	10.2	B
N16B,N17^c^ (97.4)	R37	10.5–11.4	B
N30	R52	12.5	B
N5	NAT045	22.6	C (Cc)
N62,N67^c^ (99.5)	C025	25.3–25.7	C (Cc)
N63	C024	25.6	C (Cc)
N61	NAT045	26.5	C (Cc)
N42,N45^c^ (99.8)	NAT001	29.1–29.4	C (Cc)
N10,N55,N56 (96.5–100)	C024,C026	29.9–30.3	C (Cc)
N41	NAT045	30.4	C (Cc)
N43,N50,N58C^c^ (99–99.8)	NAT045	31–31.8	C (Cc)
N35,N68 (96.1)	C025	3.0–4.0	C (Ca)
N34	NAT001	3.2	C (Ca)
N22,N33,N38,N39^c^ (99.3–99.8)	C025	15.1–15.8	C (Ca)
N1	C026	20.9	C (Ca)
N24,N25,N28,N32^c^ (99.5–100)	QPM	23.5–24.2	C (Ca)
N27,N52 (96.8)	C024,NAT045	28.7–29.1	C (Ca)
N4,N8,N21 (99–99.8)	NAT045	29.5–30.5	C (Ca)
N36	QPM	29.7	C (Ca)
N46	NAT001	30.4	C (Ca)
N7	NAT045	31.5	C (Ca)

a Subspecies classification based on local 5′UTR sequence variation (See Table 3 and Figs. 1 and 2).

b Strains closely related with more than 96% identity.

c Strains isolated within 3 months.

To characterize and classify further the Chinese HRV strains, 5′UTR sequences were considered (Table 3, Fig. 2). They were compared to the 5′ UTR of all 101 reference HRVs, to those of 26 new strains identified in children with respiratory illness in Wisconsin (indicated as W) [15], and to those of other recently identified HRV-C strains [14]. Pairwise nucleotide divergence between the three HRV species was 0.7–64.3%, and a limit of <9% divergence between genotype pairs was chosen for similar genotype assignment in one species [15]. New genotypes were identified when they had 9–30% pairwise nucleotide divergence from the nearest serotype in the same species (Table 3). Fifty-five HRVs shared >94.4% nucleotide identity with strains already identified, and 11 HRVs showed 9.5–20.9% nucleotide divergence with the nearest known HRVs. They may represent newly discovered genotypes. These strains clustered with HRV-A (N6) or HRV-C species (N4, N8, N21, N62, N63, N67, N24, N25, N28 and N32) (Table 3).

**Figure 2:**
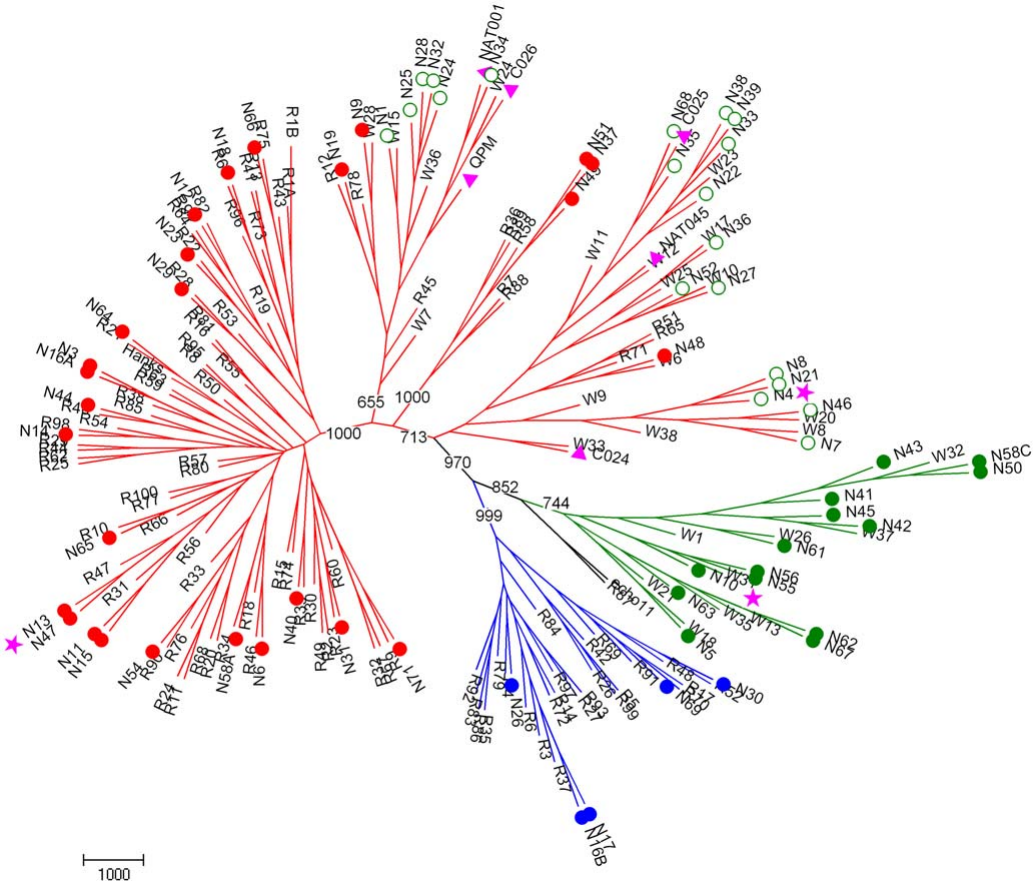
Phylogenetic tree depicting relationships between known and novel HRVs based on 5’UTR analysis. Grouping of HRV reference serotypes (designated R#), previously published novel HRV strains (designated by pink triangles), novel strains from Wisconsin [15] (designated W#), and novel viral strains detected in our clinical samples (designated by N# and circles) was based on 285 nt in the 5’UTR. Strains fully sequenced in this study are designated by a pink star. See additional legend in Figure 1.

**Table 3:** Characterization of 66 HRV strains in clinical specimens based on sequences in the 5′UTR.

Clinical HRV strains	Nearest HRV strain	Divergence with the	species (subspecies)^a^
(% nt identity)^b^		nearest HRV (% nt)	classification
N64	R21	1.4	A
N9	W28	1.4	A
N19	R12	1.8	A
N12^c^	R82	2.1	A
N3,N16A (100)	R38	2.2	A
N65	R10	2.5	A
N40	R39	3.2	A
N14	R98	3.3	A
N29	R28	3.3	A
N11,N15 (98.2)	R31	3.6–4	A
N37,N49,N51 (99.3–99.7)	R58	3.6–4.3	A
N44^c^	R40	3.7	A
N71	R9	3.7	A
N54	R90	4	A
N66	R75	4	A
N23	R22	4	A
N13,N47 (98.2)	R47	4–4.4	A
N18	R61	4.3	A
N48^c^	W6	4.3	A
N31	R23	4.4	A
N58A	R34	5.6	A
N6	R46	9.5	A
N69	R17	1.4	B
N30	R52	2.5	B
N26	R4	3.7	B
N16B	R37	4	B
N17	R3	4.8	B
N5	W18	1.1	C (Cc)
N10,N55, N56 (97.8–98.9)	W31	0.7–1.1	C (Cc)
N42,N45 (99.3)	W37	1.4–2.1	C (Cc)
N41,N43, N50,N58C (93.4–99.7)	W32	1.4–5.8	C (Cc)
N61	W26	3.6	C (Cc)
N62,N67 (99.3)	W35	10.8–11.7	C (Cc)
N63	W35	20.9	C (Cc)
N7^c^	W8	0.7	C (Ca)
N1^c^	W15	1.1	C (Ca)
N34^c^	NAT001	1.1	C (Ca)
N35,N68^c^ (97.9)	C025	1.4	C (Ca)
N22,N33,N38,N39^c^ (99–99.7)	W23	1.4–2.1	C (Ca)
N27,N52^c^ (96.2)	W10	1.8–4.4	C (Ca)
N36^c^	W17	1.8	C (Ca)
N46^c^	W20	2.1	C (Ca)
N24,N25,N28,N32^c^ (99.6–100)	W24	10.6	C (Ca)
N4,N8,N21^c^ (100)	W8	13	C (Ca)

a Subspecies based on phylogenetic analysis (Figs. 1 and 2) and on local recombination in 5′UTR (see Fig. 3).

b Strains closely related with more than 93% identity.

c These strains clustered differently when based on VP4/VP2 sequences (see Table 2 and Figs. 1 and 2).

Most surprisingly, 20 of the 34 strains classified as HRV-C by comparative analysis of VP4/P2 sequences (Table 2) were related more closely to HRV-A strains when their 5′UTRs were analyzed, and showed incongruent clustering in phylogenetic trees (Figs. 1 and 2). The nucleotide sequences in the 5′UTR of these strains were related closely to those of formerly identified QPM, NAT001, NAT045, C024, C025 and C026 HRV-C strains, and to some of the W strains recently identified as HRV-A [15] (Fig. 2). However, our 20 strains clustered together with these six strains in two major branches in the phylogenetic tree constituted a subspecies of HRV-C called HRV-Ca (Table 3, Figs. 2 and 3). The fourteen other HRV-C strains formed another unique branch of HRV-C subspecies, called HRV-Cc, which clustered differently from other species of HRV-A and HRV-B, and from subspecies HRV-Ca in the phylogenetic tree based on 5′UTR sequences (Table 3; Figs. 1 and 2). These strains were related closely to some W strains of HRV that were classified as HRV-C [15] (Fig. 2, Table 3)

**Figure 3:**
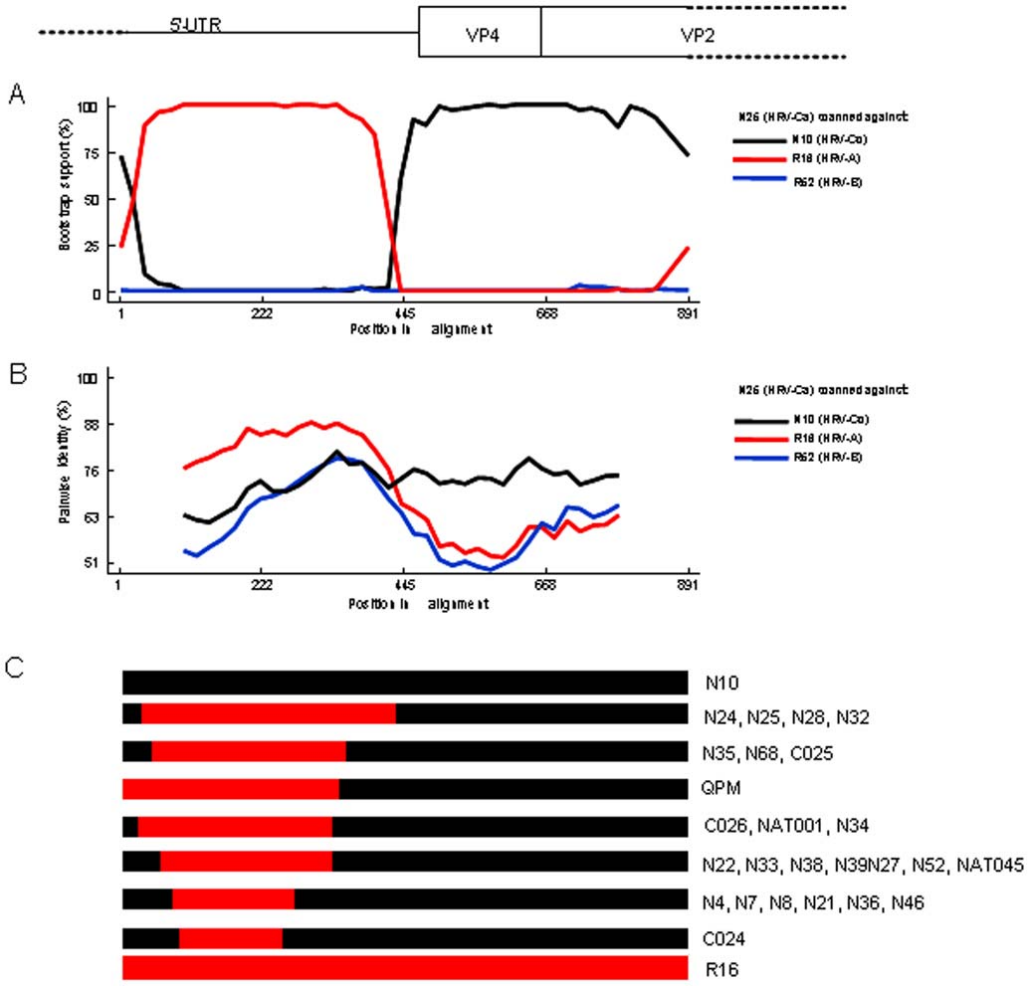
Analyses of recombination events in 5’UTR-VP2 partial sequences of HRVs. Bootscanning analysis (A) and pairwise identity (B) of N25 strain (HRV-Ca) with other strains representative of HRV-A (R16) and HRV-B (R52) species, and HRV-Cc (N10) subspecies. C: Diagram of 5’UTR-VP2 sequences of strains from HRV-Ca subspecies indicating approximate sizes and sites of recombination between HRV-A (red) and HRV-Cc (black) strain sequences.

### Recombination in the 5′ UTR

In order to characterize more precisely the differences observed in the 5′UTR between HRV-Ca and HRV-Cc subspecies, and to localize possible recombination sites in the 5′UTR of the genome of HRV-Ca subspecies, bootscanning and similarity plot analyses were conducted in the gene fragment of 868 nt that included the 5′UTR and adjacent capsid genes. HRV-Ca nucleotide sequences were scanned against sequences of N10, R16 and R52, which are considered as representative strains of HRV Cc subspecies and HRV-A and HRV-B species, respectively. Stretches of nucleotide sequences that were closer to HRV-A (R16) than to HRV-Cc (N10), flanked by sequences related to HRV-C could be detected in the 5′UTR of HRV-Ca strains, as exemplified with HRV-Ca N25 strain (Fig. 3a and b). These HRV-A-related nucleotide stretches were thus flanked by putative recombination sites. These sites were located differently among the HRV-Ca strains, delimiting HRV-A-related stretches that ranged from 150 to 400 nt in length (Fig. 3c). While variable among the strains, the identified recombination sites were all located inside the 5′ UTR and none of them was identified in the downstream VP4/VP2 coding sequence. HRV-A-related nucleotide sequences and putative recombination sites were also found in the 5′UTR of the previously described HRV-C strains C024, C025, C026, NAT001, NAT045 and QPM (Fig. 3). The results corroborated the clustering observed in the phylogenetic tree based on 5′UTR sequences (Fig. 2), since strains gathered in the same HRV-Ca subcluster (for example N24, N25, N28 and N32, or N4, N7, N8, N21, N36 and N46) displayed the same recombination pattern. These subclusters revealed different recombinant lineages, each of which originated from independent recombination events.

### Comparative analysis of full-length genomes of HRV-A, HRV-B and HRV-C species

In order to further characterize the genome of the HRV-Cc subspecies, for which no full-length sequence was yet available, we sequenced the remaining genes that covered the whole coding sequence and 3′UTR of N10 strain, which was chosen as the representative of this subspecies (Tables 2 and 3). The full-length N10 genome sequence was compared to those of the HRV-Ca subspecies strains C024, C025 and C026, and to that of N4 strain, which was sequenced as the representative of the HRV-Ca subspecies. The genome sequences were also compared to those of the HRV-A strains N13 and R44, and to the HRV-B strains R14 and R52 (Table 4). The full-length nucleotide sequence of N10 strain contained 7111 nt, excluding the poly(A) tract, which was shorter than sequences from HRV-A and HRV-B strains, but similar to those of HRV-Ca strain N4 and other related strains (C024, C025 and C026). The 2144 aa lengths of the polyprotein and of each of the individual proteins of N10 were slightly different from those of HRV-A and HRV-B species, but similar to those of other HRV-C strains. The most divergent amino acid length between HRV was observed for the VP1 protein that was shorter in HRV-C species (Table 4). The unique putative cleavage (M/S) site between VP4 and VP2 protein identified previously for QPM, C024, C025 and C026 strains [20] was also observed for N10 and N4 strains. It was different from those of the HRV-A strains N13 and R44 (Q/S), and from those of the HRV-B strains R14 and R52 (N/S) (data not shown).

**Table 4:** Comparative study of HRV genomes and individual proteins.

Strain	Species	Genome length	5′ UTR	3′UTR	ORF	Viral proteins											No Genbank
		(nt)	(nt)	(nt)	(aa)	VP4 (aa)	VP2 (aa)	VP3 (aa)	VP1 (aa)	2A(aa)	2B(aa)	2C(aa)	3A(aa)	3B(aa)	3C(aa)	3D(aa)	
R44	A	7123	612	49	2154	69	266	238	281	142	95	322	77	21	183	460	DQ473499
N13^a^	A	7136	612	50	2158	69	265	238	286	142	95	322	77	21	183	460	CQ223229
R14	B	7212	628	47	2179	69	262	236	289	146	97	330	85	23	182	460	K02121
R52	B	7216	624	43	2183	69	262	236	293	146	97	330	85	23	182	460	FJ445188
N10^a^	C	7111	641	43	2143	67	262	235	272	142	99	326	75	22	183	460	CQ223228
N4^a^	C	7107	619	44	2148	67	262	236	276	142	99	326	75	22	183	460	CQ223227
C024	C	7099	615	52	2144	67	261	235	274	142	99	326	75	22	183	460	EF582385
C025	C	7114	616	42	2152	67	264	239	275	142	99	326	75	22	183	460	EF582386
C026	C	7086	611	49	2142	67	262	233	275	142	97	325	76	22	183	460	EF582387

a Strains of this study.

nt: nucleotides; aa: amino acids; ORF: open reading frame; UTR: untranslated region.

Alignment of the VP1 amino acid sequence of HRV-Cc strain N10 with those of other HRV-A and HRV-B species and HRV-Ca subspecies, designated in Table 4, showed structural features typical of HRV-C species [16], [20], [25] (data not shown). In particular, footprints including deletions in the BC, DE and HI loops and conserved amino acids potentially involved in Inter-Cellular Adhesion Molecule 1 (ICAM-1) receptor binding [7], [11], [20], [24] were conserved within the HRV-C species (data not shown).

**Figure 4:**
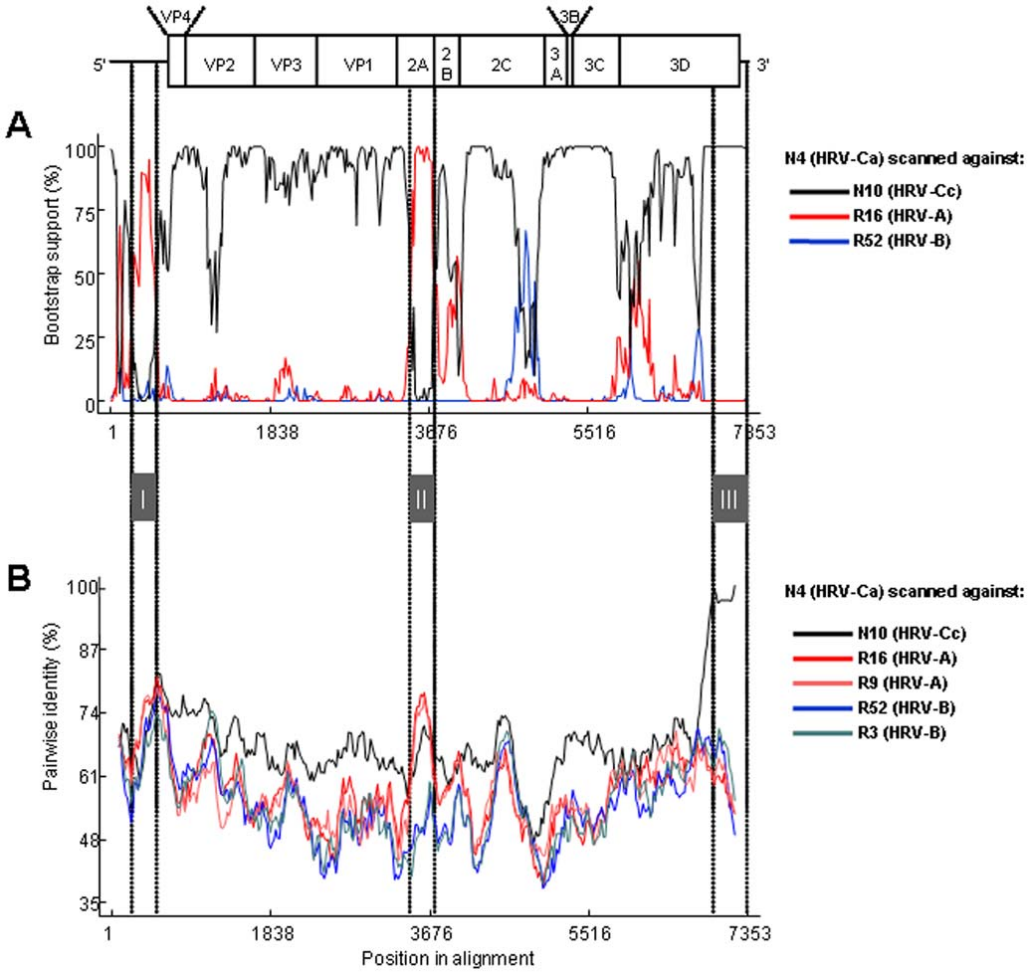
Analyses of recombination events in N4 isolate full-length genomic sequence. A: Result of manual bootscanning of N4 genome against several viruses. B: Pairwise identity of N4 with several viruses. Grey boxes indicate possible recombination sites.

Bootscanning and similarity plot analysis conducted on the genomic sequences of N4 (HRV-Ca), N10 (HRV-Cc), R16 (HRV-A) and R52 (HRV-B) confirmed that N4 featured a 5′UTR sequence that was related to the R16 sequence (stretch I), followed by a capsidic sequence related to the N10 sequence. N4 non-structural sequence (2A to 3′UTR) was related more closely to N10 than to R16 and R52 sequences. However, in stretch II (nt 3300–3500 according to N4 numbering), N4 strain (HRV-Ca) was closer to R16 (HRV-A) than to R52 (HRV-B) or N10 (HRV-Cc), which resulted in high bootstrap values between N4 and R16 2A sequences (Fig. 4). This may have been the result of a recombination event that occurred in the 3′ part of the 2A-encoding sequence of the parental strain N4, and which involved an HRV-A strain. Nevertheless, the HRV-A parental strain or ancestral strain could not be identified since the closest HRV-A 2A nucleotide sequence available was <80% identical to that of N4 in this region.

**Figure 5:**
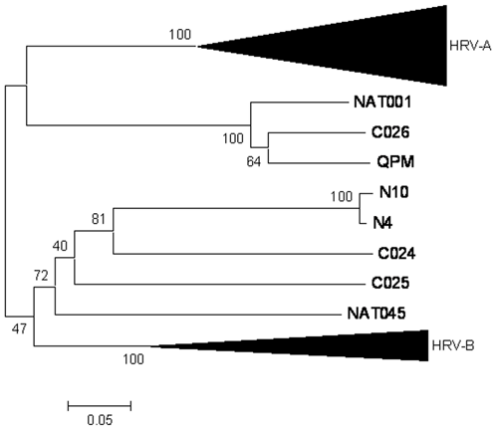
Phylogenetic tree based on the 3’ terminal part of the viral genomes (nt 6650 to end, according to N4 numbering). Dark triangles represent HRV-A and HRV-B sequences clustering together.

In contrast from nt 6,550 to the 3′ end (stretch III in Fig. 4), the N10 strain genome was found to be closely related to that of N4, with nucleotide identity >98%. This result is corroborated in Figure 5, which shows a phylogenetic analysis of the 3′UTR sequences of N10 and N4 compared to those of HRV-Ca subspecies and HRV-A and HRV-B species. This suggests that N4 and N10 strains share a common recent ancestor through recombination.

### Clinical outcome from HRV strains isolated from pediatric outpatients

Among the pediatric patients, 46 were males and 18 females, and their age ranged from 5 months to 14 years. The majority of HRV infections were diagnosed between 2 and 6 years of age (84.6%). Bronchitis (73.4%) and pneumonia (26.6%) were highly prevalent in children with comparable incidence in HRV-A and HRV-C infections (Table 5). Moreover, the ratio of pneumonia over bronchitis (36.2%) was comparable to that in the whole cohort of 827 children (40.7%). Only one child among the 64 HRV-positive patients had asthma and was co-infected with HRV-C, influenza A virus (IAV) and respiratory syncytial virus (RSV) (Table 5), whereas 100 of the 827 patient were diagnosed with asthma. HRVs were isolated throughout the 2 years, with a predominance of HRV-C viruses in the cold season (Table 5). Interestingly, different HRV genotypes were detected within the same period (for example, N1 and N4, N9 and N11/N12, N44/N48 and N51, and N55/N56 and N62/N67), with a larger diversity and distribution of individual or paired HRV-A genotypes compared to HRV-C strains, which clustered in closely-related genotypes (Fig. 2, Tables 2 and 3). Conversely, N4 and N21 strains of samples collected at 10 months interval showed 99.8% identity (Fig. 2).

**Table 5:** Clinical seasonal, and virological characteristics by HRV group.

	HRV species				
	A	B	C	A+B^a^	A+C^b^
Number of cases	25	4	33	1	1
Symptoms					
Bronchitis	18	2	26		1
Pneumonia	7	2	7	1	
Season					
Spring	3	0	7		1
Summer	9	1	4		
Autumn	6	3	7	1	
Winter	7	0	15		
Single Infection	19	1	22		
Co-infection	6	3	11	1	1
PIV1	2				1
ADV	1		2(1^c^)		
BoV	2		4(2^c^)		
RSV		1	6(5^c^)	1^c^	
HEV	1	2			
HMPV			1		
IAV			1^c^		

a Patient co-infected by two HRV-A and HRV-B strains, and RSV.

b Patient co-infected by HRV-A and HRV-C strains, and PIV1.

c Cases with triple infection : RSV/IAV/HRV-C, RSV/ADV/HRV-C.

RSV/BoV/HRV-C, RSV/BoV/HRV-AC, RSV/HRV-A/HRV-B.

Single HRV infection was diagnosed in 42 children and co-infections were identified in 22 patients (Table 5), with 17 double and five triple infections. The viruses most often identified in HRV co-infection were RSV (six cases) and human bocavirus (HBoV; four cases), and two patients were co-infected with HRV, HBoV and RSV (Table 5). There was no difference between HRV-Ca or HRV-Cc subspecies and any of the clinical or epidemiological data (data not shown).

## Discussion

In this report, we looked for HRVs in a 2-year collection of NPSs from children with ARI visiting a district hospital in Shanghai, and found a high diversity of HRV strains that belonged to different species and genotypes. We characterized by RT-PCR and sequenced 66 HRVs, among them 27 HRV-A, five HRV-B, and 34 HRV-C strains. When sequencing the VP4/VP2 region of the HRV genome, several recent studies have identified new strains of viruses from children and adults with ARI, asthma, or otitis, which are clustered differently from HRV-A and HRV-B, and have been classified into a novel HRV-C species [7], [8], [17], [18], [19], [20], [21], [25], [26], [27], [28]. Other groups have also identified novel HRV-C strains by sequencing the VP1 gene [29] or the 5′UTR [14], [15] . The different sizes and locations of the regions amplified in the HRV genomes renders difficult comparative genetic analysis. Recently, Palmenberg et al. (2009) have finalized the full-length genome sequences of all HRV-A and HRV-B reference strains, and identified structural features of these two species and the novel HRV-C species [16]. In our study, we identified 34 HRVs (51.5%) that clustered differently from HRV-A and HRV-B in a phylogenetic tree that was established on the basis of VP4/VP2 sequences, which were related to recent strains classified in the novel HRV-C species (Fig. 1, Table 2). Fourteen HRV-C strains (41.2%) segregated from the other 20 strains (58.8%) that were closely related to HRV-A in their 5′UTR sequence (Fig. 2). This led us to propose a classification of two HRV-C subspecies, HRV-Cc and HRV-Ca. In previous studies targeting the 5′UTR of HRVs, Lee et al. (2007) have identified nine novel HRVs among 103 HRVs from Wisconsin (19.4%), which segregated from HRV-A and HRV-B and were classified as HRV-C [15]. These strains clustered with our field strains within the HRV-Cc subspecies. Moreover, 17 strains that clustered with HRV-A, and had 12–35% pairwise nucleotide divergence from the nearest reference serotype [15], clustered within the two major branches of HRV-A and HRV-Ca strains (Fig. 3). Therefore, we cannot ensure that some of the 17 strains were HRV-A or HRV-Ca strains. Kiang et al. (2008) have identified five novel HRVs out of 24 clinical samples (20.8%), which segregated from HRV-A and HRV-B, and were classified as HRV-C, and three additional strains (12.5%) that also clustered with QPM, C024, C025, C026, NAT001 and NAT045 [14] (Fig. 2). However, the field HRV strains of these previous studies were sequenced using a 5′UTR that did not match fully our sequence and that of Lee et al. (2007) [15], and could not be included in the present study for comparative analysis. Interestingly, the five strains identified in California in 2007 [14] and N42 and N45 from our study were closely related to strain W37 isolated in Wisconsin in the late 1990s [15], and to NAT001 isolated in the winter of 2004 in California [18], which confirms that similar genotypes of HRV-Ca are widespread [17].

The strains of HRV-C species identified in the present study were characterized by analyzing the 5′UTR, VP4, and part of VP2 (Fig. 3). This approach showed the advantages of covering only 5′NCR, VP4/VP2, VP1 or 3D genome fragments. Analyzing sequences that covered the 5′UTR and the downstream VP4/VP2 capsid region allowed identification of co-infections when several clones were sequenced, and helped to locate the recombination sites in strains of the HRV-Ca subspecies. Thus, this region of the genome may be useful for building a database of the novel strains that are circulating worldwide.

The genome of HEVs is subject to frequent recombination [30], [31], [32], [33], [34], [35], [36], with interspecies exchanges observed in the 5′UTR [37]. Palmenberg et al. (2009) have observed intraspecies recombination in three HRV-A, with structural characteristics and phylogenetic evidence that suggests a novel clade D classification [16]. Tapparel et al. (2009) observed phylogenetic incongruities in 5′ UTR, VP1 and 3CD sequences of two clinical isolates of HRV-A related to recombination [38]. We observed incongruent clustering of N12, N44 and N48 strains of HRV-A species in the phylogenetic trees based on the 5′UTR or the VP4/VP2 regions of their genomes (Figs. 1 and 2, Table 3), which suggests intraspecies recombination in the 5′UTR.

We observed one co-infection with HRV-A and HRV-B (N16A and N16B), one with HRV-A and HRV-C (N58A and N58C), and three co-infections of HRV-A and HRV-B with HEVs that may favor recombination events. Previous comparison of genome sequences between 34 HRVs showed only limited recombination events and a pattern of genetic diversity lower than that observed with other picornaviruses [25]. The presence in HRV-C subspecies of sequences that share 90.5–98.6% identity with HRV-A strains (Table 3) suggests that recombination events occurred between HRV-C and HRV-A. Bootscanning of the 5′UTR of HRV-C strains also showed different sites and lengths of recombination (Fig. 3c), which suggested that there were several independent events that led to several groups of HRV-Ca genotypes, which formed clusters in the phylogenetic tree (Fig. 2).

Comparative analysis of the full-length nucleotide sequences of two field strains of different HRV-C subspecies (N4 and N10) with those of other HRV species suggested that multiple interspecies recombination events occurred in the 5′UTR and in the NS2A protein gene, and that recombination also occurred in the 3′UTR between N4 and a strain close to N10. These findings are in agreement with those observed for other HEVs, for which recombination events in the capsid-encoding sequence are very rare, probably because of structural constraints that restrict the functioning of chimeric capsids [31]. This result appeals for the full-length genome sequencing of the major representatives of the HRV-C species, in order to establish a clear understanding of the evolution and classification of the novel virus into subspecies. Comparison of the coding sequences of N10 HRV-Cc with other strains of HRV-Ca subspecies [20], including our field strain N4, showed high similarities in the lengths of the 11 proteins, their cleavage sites, and the structural features of VP1. These characteristics and the absence of growth in cell culture, noted in our laboratory and by others (data not shown), support the classification of the novel strains into a unique HRV-C subspecies.

Our clinical specimens all originated from NPSs from pediatric outpatients. The remarkable outcome of the study is the large diversity of genotypes that has circulated in a relatively small group of people in a district of Shanghai during a 2-year observation. Although some clusters of similar genotypes in a limited period of time were observed, co-circulation of different genotypes and HRV species and subspecies, and co-infections with two HRV species were observed. The prevalence of the novel HRV-C in our specimens (4.1%) differed from previous studies that associated the prevalence of the novel variant with severe disease outcomes, which ranged from influenza-like illness or infection of the low respiratory tract [17], [28] to asthma exacerbation, bronchiolitis, and febrile wheeze [7], [8], [15], [18], [20], [21], [29], [39]. All our patients showed bronchitis or pneumonia, with no etiological correlation with any of the species or subspecies of HRV. Only one patient co-infected with HRV-C, IAV and RSV was diagnosed with asthma among the HRV-positive patients (1.6%), whereas 100 of the 827 children had asthma (12%). The difference observed with previous studies, 44.6% [8] and 12% [29] asthma in HRV-positive patients, may be related to the criteria for enrolment. Moreover, none of the patients in our study were hospitalized, which makes comparison with hospitalized children difficult [20], [24]. Another criterion to consider in the trend to correlate clinical symptoms with HRV infection is the presence of co-infecting pathogens. In our study, four strains of HBoV and six strains of RSV (17.6%) were identified in association with HRV-C (11.7%). HBoV and RSV are common viruses diagnosed in ARI, which are often associated with HRV [40], [41], and HBoV was identified in >50% of children co-infected with HRV [20]. Nevertheless, the incidence of HBoV in ARI and in severe outcomes remains elusive [42]. More studies need to be carried out on large numbers of samples from severe and mild diseases, to identify any obvious role of HRV sequence diversity and association with other pathogens in disease severity. Since a large diversity of recombination in HRVs has become obvious, we must be aware of the occurrence of novel HRVs that may become highly virulent.

## Materials and Methods

This study was approved by the ethical committee of Shanghai Nanxiang Hospital and written informed consent was obtained from the parents of the children.

### Specimens and viruses

Clinical specimens (*n* = 827) from NPSs were collected from children under 14 years old, who experienced a lower respiratory tract infection, and who were consulting the pediatric department of Shanghai Nanxiang Hospital during the period October 2006 to October 2008.

### Multiplex RT-PCR assay

Total RNA was extracted from NPS specimens using QIAamp viral RNA Mini Kit (Qiagen, Hilden, Germany), and stored at −80°C. RNA was amplified using the Qiagen One Step RT-PCR Kit. A five-tube mRT-PCR was used for virus identification as previously described [43], [44]. Tube 1 targeted IAV, influenza B virus, RSV, and human metapneumovirus; tube 2, parainfluenza viruses 1 to 4; tube 3, HRV and influenza C virus; tube 4, human coronaviruses (HCoVs) 229E-HCoV, OC43-HCoV, NL63-HCoV and HKU1-HCoV; and tube 5, adenovirus and HBoV. Amplified products were analyzed in 0.5 µg/ml ethidium bromide/2% agarose gel.

Samples that showed positive results for HRV were amplified again using specific primers P1-1F and VP4/2R, located in the 5′UTR and VP2 gene, respectively (Table 1). One strain of HRV-C (N1) could not be amplified using the P1 and VP2 extreme primers and was amplified using primers in 5′UTR and VP4/VP2, respectively (Table 1). In brief, 2.5 µl of extracted RNA was mixed with 5× buffer and 0.4 mM dNTPs, 0.2 µM of each of the primers, and 1 µl of enzyme mix, and diethylpyrocarbonate-treated ultrapure water was added to a final volume of 25 µl. Amplification programs included reverse transcription at 50°C for 30 min, inactivation at 95°C for 15 min, followed by 40 cycles at 94°C for 30 s, 50°C for 30 s, 72°C for 70 s, and final extension at 72°C for 10 min. The amplified DNA products were detected by ethidium bromide–agarose gel electrophoresis. The lengths of P1-VP2, VP4-VP2 and P1-P3 amplicons were 924, 559 and 390 nt, respectively. DNA products were extracted from agarose gels by using QIAquick Gel Extraction Kit (Qiagen), and were ligated into pMD20-T vector (Takara Biotechnology, Dalian, China), and at least two recombinant plasmids were sequenced in Biosune Sequence Company and Life Biotechnology in Shanghai, China. Sequences of different clones of N16 and N58 showed identities for either HRV-A or HRV-B strains. More plasmids were sequenced for these strains to confirm that the two patients were originally co-infected with two different HRV species.

### Complete genome sequencing

Sequences of three complete genomes of HRV were obtained for strains N4 (reference R3061207002 collected on December 7, 2006), N10 (R3070614001 collected on June 14, 2007) and N13 (R3070719007 collected on July 19 2007). Primers used for the amplification of viral genomes were designed after multiple alignments of sequences from the genomes of different HRVs available in GenBank (Table 1). Overlapping amplified DNA products were obtained after PCR of cDNA that was obtained using oligodT and a Transcriptor High Fidelity cDNA Synthesis Kit (Roche, Mannheim, Germany), following the manufacturer's protocols. Briefly, 10.4 µl viral RNA was mixed with 1 µl oligodT and heated at 65°C for 10 min, and then kept on ice for 2 min. After addition of 4 µl 5× buffer, 0.5 µl Protector RNase Inhibitor, 2 µl dNTPs, 1 µl DTT, and 1 µl RT enzyme, the reaction was incubated at 50°C for 1 h, inactivated at 85°C for 5 min, and stored at −20°C.

Amplification of a 3D region of N4, N10 and N13 HRV strains was carried out by nested-PCR using Takara EXTaq (Takara Biotechnology) and specific primers (Table 1), for 35 cycles of 30 s at 94°C, 30 s at 55°C, and 70 s at 72°C. To amplify VP1 (upstream of 2A) sequences of N4 and N13 strains, nested PCR was carried out using Takara LATaq with GC buffer I(Takara Biotechnology), specific primers (Table 1), and incubation for 35 cycles of 30 s at 94°C, 30 s at 60°C, and 4 min at 68°C. The fragment VP2–3D of N10 HRV strain was obtained by semi-nested PCR and specific primers VP2 F, and 3D inner and outer reverse primers (Table 1), using Takara LATaq with GC buffer II, for 35 cycles of 30 s at 94°C, 30 s at 60°C, and 6 min at 68°C. The terminal part of the whole genome was obtained by rapid amplification of cDNA ends using 5′/3′ rapid amplification of the cDNA kit, following the manufacturer's protocol (Roche). To perform 5′ terminal RACE, 4 µl of 5× cDNA buffer, 2 µl dNTPs, 1.25 µl specific primer 1 (10 µM), 9.2 µl RNA, 1 µl control primer neo1/rev (12.5 µM), 1 µl control RNA, 1 µl RT enzyme, and 0.6 µl RNase inhibitor (Roche) were mixed and incubated for 55°C for 60 min, followed by inactivation at 85°C for 5 min, and stored on ice. The product was purified using the Qiagen PCR Purification Kit and eluted with 30 µl deionized distilled water. A polyA tail was added to the cDNA, by mixing 9.5 µl DNA with 1.25 µl 10× reaction buffer, 1.25 µl (2 mM) dATP, and after incubation at 95°C for 3 min, the reaction was chilled on ice for 2 min. After addition of 0.5 µl terminal transferase, the reaction was incubated at 37°C for 30 min, inactivated at 70°C for 10 min, and kept on ice. Nested PCR was performed by using the Expend High Fidelity PCR kit (Roche). A mixture of 2.5 µl poly-dA-tailed cDNA, 0.5 µl oligodT-anchor primer 37.5 µM, 0.62 µl SP2 primers (10 µM) (Table 1), 0.5 µl control neo2/rev primer (12.5 µM), 0.5 µl dNTP, 0.35 µl enzyme, 2.5 µl 10× buffer, and 18 µl ddH_2_O was incubated for 40 cycles of 30 s at 94°C, 30 s at 60°C, and 30 s at 72°C. To perform 3′ terminal RACE, the method was similar to normal two-step RT-PCR using 3D inner and outer F primers (Table 1).

### Sequence alignment, phylogenetic analyses and recombination analysis

DNA sequences used for P1-P2 gene analysis were based on HRV-16 nt 178–462 and those used for VP4/VP2 gene analysis were based on HRV-16 nt 626–1045. Multiple sequences were aligned using Clustal X [45]. The multiple-sequence alignment was subjected to phylogenetic analyses using programs in the PHYLIP package (v3.6). Bootstrap analysis was performed using SEQBOOT, with a replicate number of 1000. Then, DNADIST and NEIGHBOR were used to obtain distance matrices with the F84 parameter, and a transition/transversion ratio of 4. Consensus trees were computed by CONSENSE, and then re-rooted with RETREE. The final tree was visualized and edited with MEGA version 4 [46].

Recombination analysis was carried out by using Recombination Detection Program v.3.22. Manual bootscanning was performed by using the Juke-Cantor algorithm and the neighbor-joining method [47], with a window size of 200 nt, a step size of 20 nt and 100 replicates. Pairwise identities between sequences were determined with SimPlot software method [48],with a window size of 200 nt and a step size of 20 nt. Pairwise homology matrices were obtained by using CLC Combined Workbench 3.0 software (CLC bio, Aarhus, Denmark).

### Nucleotide sequence accession numbers

The original P1-VP2 sequences described in this study were deposited in GenBank under accession nos. GQ223119 to GQ223136. The VP4-VP2 sequences were deposited under the nos GQ223137 to GQ223181, and the P1-P2 sequences under the nos GQ223182 to GQ223226. The full length genomes sequences of N4, N10 and N13 strains were deposited under the nos. GQ223227, GQ223228, GQ223229, respectively.
